# Supplementary material for: Influence of electronic polarization on the binding of anions to a chloride-pumping rhodopsin
Source: Biophys J. 2023 Mar 21;122(8):1548–56. doi: 10.1016/j.bpj.2023.03.026 (PMC10147828; doi:10.1016/j.bpj.2023.03.026)
Supplement: Document S1. Figures S1–S8 and Table S1 [file mmc1.pdf]

Biophysical Journal, Volume *122*

Supplemental information

Influence of electronic polarization on the binding of anions to a chloride-pumping rhodopsin

Linda X. Phan, Victor Cruces Chamorro, Hector Martinez-Seara, Jason Crain, Mark S.P. Sansom, and Stephen J. Tucker

Biophysical Journal, Volume *122*

Supplemental information

Influence of electronic polarization on the binding of anions to a chloride-pumping rhodopsin

Linda X. Phan, Victor Cruces Chamorro, Hector Martinez-Seara, Jason Crain, Mark S.P. Sansom, and Stephen J. Tucker

Figure S1

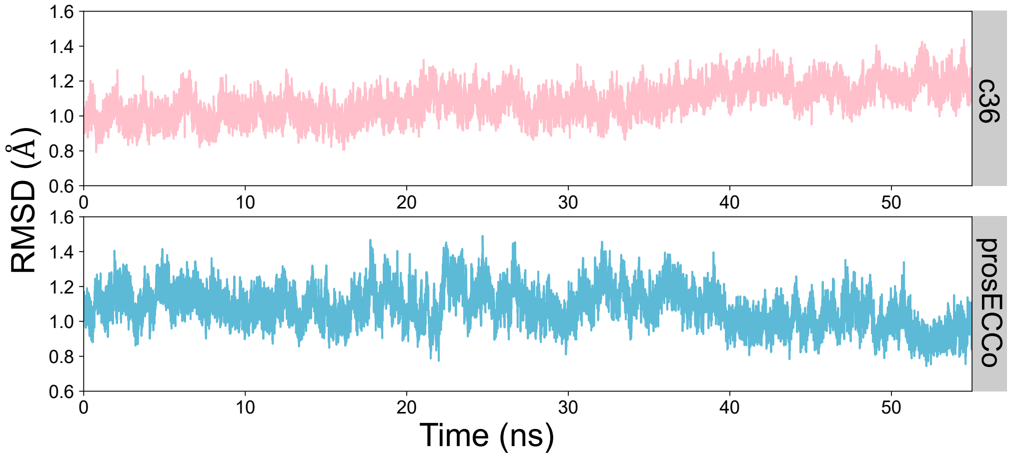

Figure S1: RMSDs of the protein backbone in simulations of the whole protein embedded in a lipid bilayer without backbone position restraints applied. The protein deviates by < 2.0 Å compared to the crystal structure and therefore justifies omitting the use of backbone restraints in simulations of the whole protein.

Figure S2

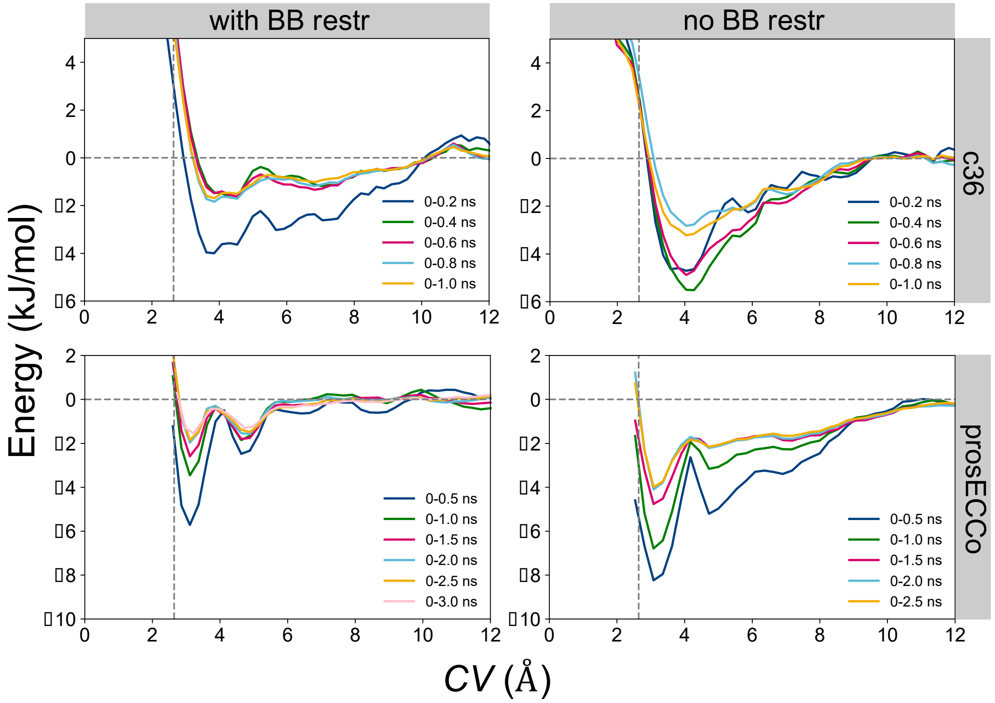

Figure S2: Cl^{-} PMF convergence analysis using the CHARMM36 (c36) and prosECCo force fields with and without the use of protein backbone restraints. Convergence analysis was performed by calculating 0.2 ns and 0.5 ns sampling blocks over the sampling time for of 1.0 ns and 2.5 ns for c36 and prosECCo respectively. The vertical grey dashed line represents the position of the crystal structure binding site in terms of *CV*.

Figure S3

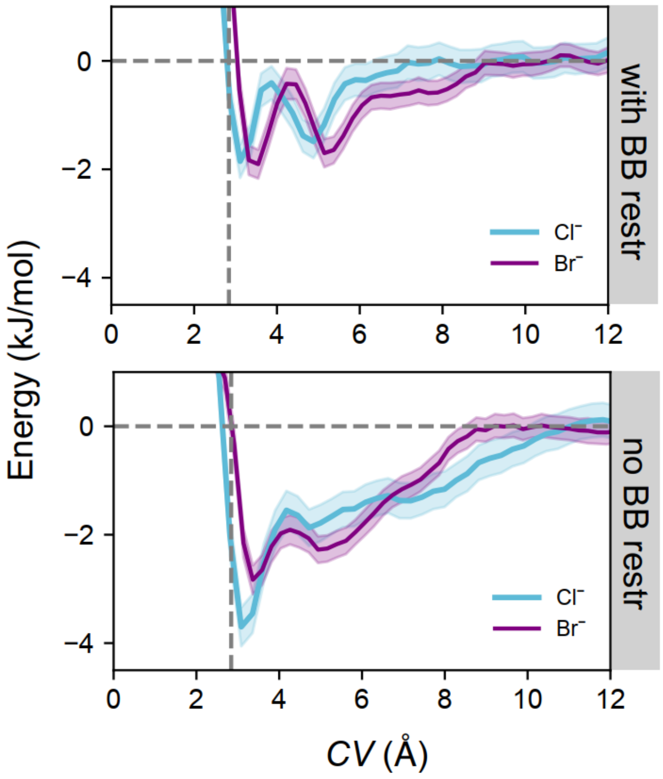

Figure S3: Potential of mean force profiles for a Cl^- and Br^- ion moving away from the binding site into bulk solution employing the prosECCo force field. Protein backbone restraints were not applied to the simulations. The horizontal grey dashed line represents the free energy of the ion in bulk solution and the vertical grey dashed line is representative of the location of the corresponding ion in the resolved crystal structure.

Figure S4

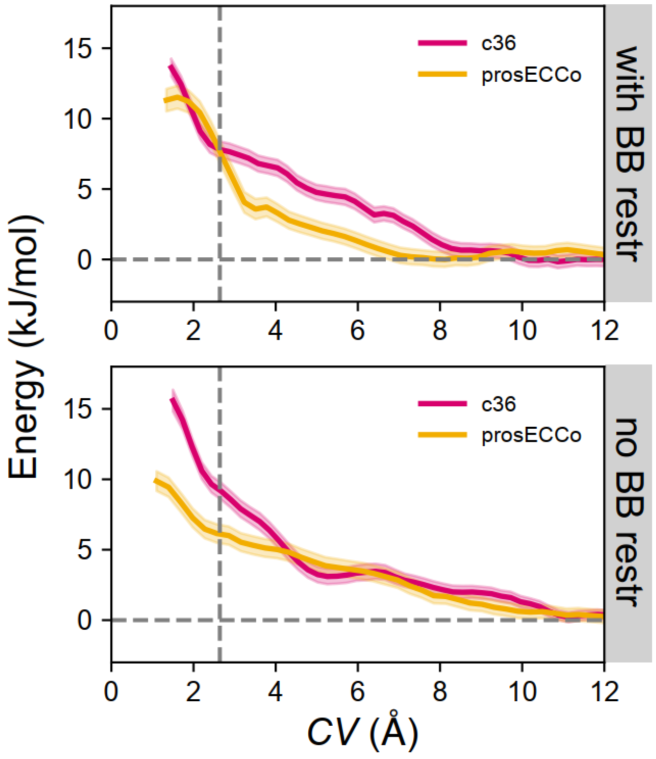

Figure S4: Potential of mean force profiles for a Na^+ ion moving away from the binding site into bulk solution with the c36 and prosECCo force fields. Na^+ does not appear to bind to the defined binding site. The horizontal grey dashed line represents the free energy of the ion in bulk solution and the vertical grey dashed line is representative of the location of the Cl^- ion in the resolved crystal structure in terms of *CV*. Samples over 1 ns per umbrella window were used in unbiasing for both force fields.

Figure S5

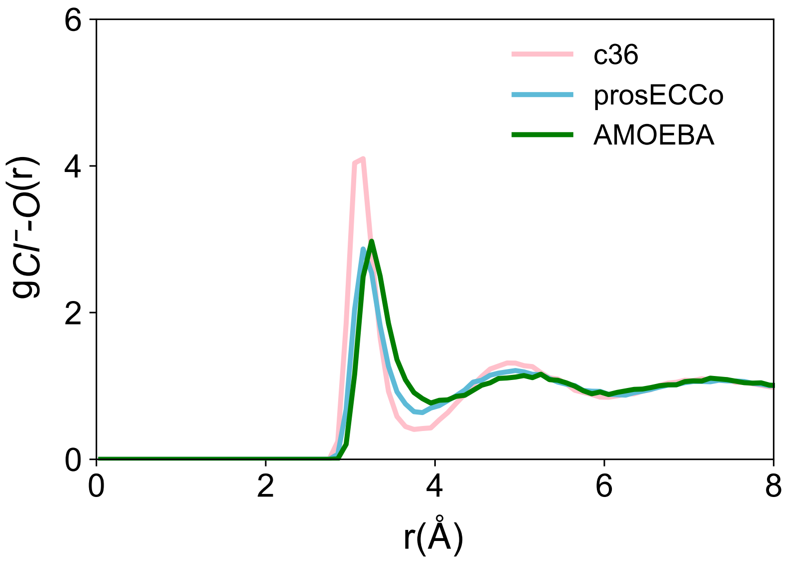

Figure S5: Radial distribution functions (RDFs), $g_{Cl^{-}\text{-}O}$(r), of water oxygen atoms around a Cl^{-} ion in bulk solution with the c36, prosECCo and AMOEBA forcefields.

Figure S6

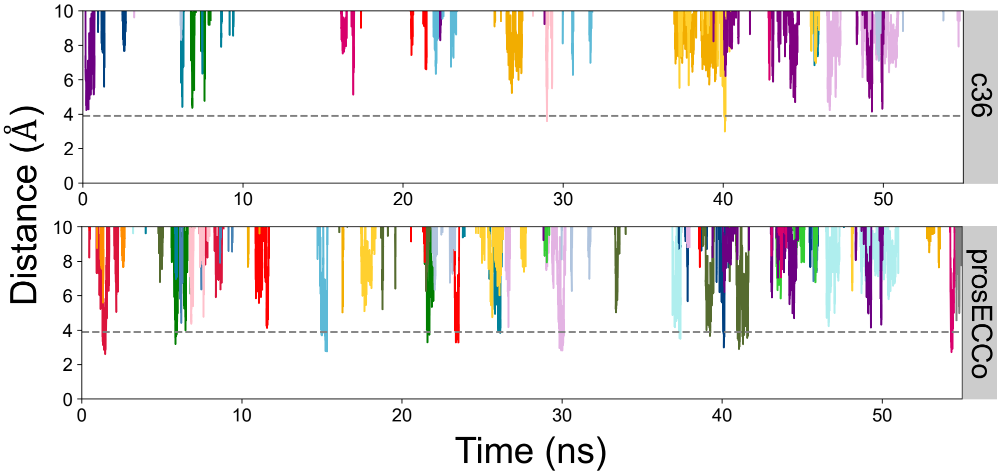

Figure S6: Ion-distance plots between Cl^- ions to an aliphatic hydrogen atom of A44 within simulations of the whole protein embedded in a lipid bilayer with the c36 and prosECCo forcefields. Each color represents an individual ion trajectory, plotting only the ions that initially come within a distance of 5 Å to the binding site.

Figure S7

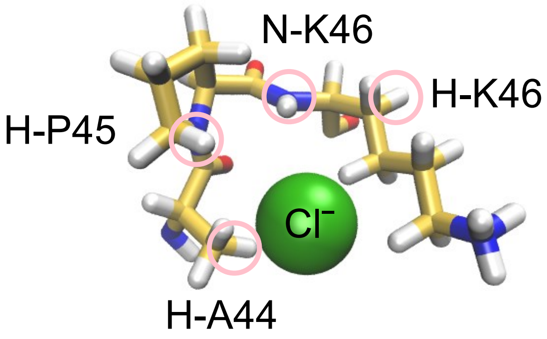

Figure S7: Snapshot of a Cl^{-} ion in the binding site. This is an example of where the K46 tail encloses the ion to form a deeper concave shape that facilitates ion binding.

Figure S8

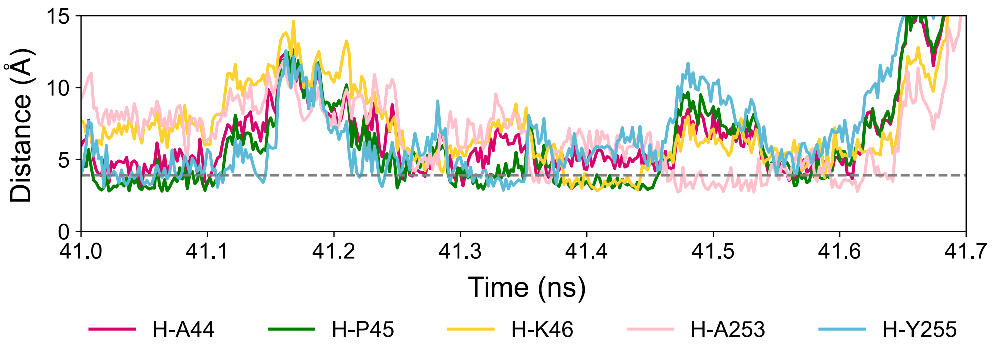

Figure S8: Ion-distance plot as a function of time for a Cl^{-} ion in the binding site and residues from a nearby loop. The ion distances were measured from simulations of the whole protein embedded in a lipid bilayer using the prosECCo force field. The Cl^{-} is bound to the site (contacts H-A44, H-P45 & H-K46) then dissociates (at ~ 41.5 ns) and forms interactions with residues from the nearby loop (H-A253 & H-Y255). Cl^{-} rebinding can be observed (at ~ 41.6 ns) and the ion is then released into bulk solution (at ~ 41.7 ns).

Table S1

System Size	Force field	ns/day
Whole Protein ~ 82300 atoms	c36	110
	prosECCo	107
	AMOEBA	N/A
Fragments ~ 12100 atoms	c36	690
	prosECCo	697
	AMOEBA	28

Table S1: Computational performance comparison of different system sizes per force field. All systems were benchmarked using 1 GPU (NVIDIA Quadro RTX8000), 1 node with 6 cores (CPUs: Intel Xeon Platinum 8268). c36 and prosECCo have very similar computational costs by running on GROMACS 2020. AMOEBA was run with the OpenMM 7.4.2 implementation which enables GPU acceleration and is considerably more computationally expensive relative to c36 and prosECCo.
